# An Eye Movement Study on the Role of the Visual Field Defect in Pure Alexia

**DOI:** 10.1371/journal.pone.0100898

**Published:** 2014-07-07

**Authors:** Tobias Bormann, Sascha A. Wolfer, Wibke Hachmann, Wolf A. Lagrèze, Lars Konieczny

**Affiliations:** 1 Neurologische Universitätsklinik Freiburg, Freiburg, Germany; 2 Kognitionswissenschaft, Albert-Ludwigs-Universität Freiburg, Freiburg, Germany; 3 Institut für Deutsche Sprache (IDS), Mannheim, Germany; 4 Università degli Studi di Trento, Scienze della Cognizione e della Formazione, Trento, Italy; 5 Universitäts-Augenklinik Freiburg, Freiburg, Germany; Universidade Federal do ABC, Brazil

## Abstract

Pure alexia is a severe impairment of word reading which is usually accompanied by a right-sided visual field defect. Patients with pure alexia exhibit better preserved writing and a considerable word length effect, claimed to result from a serial letter processing strategy. Two experiments compared the eye movements of four patients with pure alexia to controls with simulated visual field defects (sVFD) when reading single words. Besides differences in response times and differential effects of word length on word reading in both groups, fixation durations and the occurrence of a serial, letter-by-letter fixation strategy were investigated. The analyses revealed quantitative and qualitative differences between pure alexic patients and unimpaired individuals reading with sVFD. The patients with pure alexia read words slower and exhibited more fixations. The serial, letter-by-letter fixation strategy was observed only in the patients but not in the controls with sVFD. It is argued that the VFD does not cause pure alexic reading.

## Introduction

Pure alexia is a severe impairment of reading with largely preserved writing and other language skills [1]. Although the name implies preserved writing and patients usually exhibit little impairment of spoken language processing, their reading is so impaired that it affects the processing of even short words. Unfortunately, many patients with pure alexia remain unable to read fluently. The impairment usually follows from lesions to the left fusiform gyrus [2]–[4] but other associated lesions have been reported [5]–[6].

Some individuals are unable to read at all suffering from what has been called “global alexia” [4] but even those patients with a milder form of pure alexia are often unable to recognize words they had just written a few minutes ago. To process words, patients resort to a slow, serial letter-by-letter identification strategy which led to the alternative term “letter-by-letter reading”. The terms “pure alexia” and “letter-by-letter reading” have often been used interchangeably. The former highlights preservation of writing and other language functions while the latter refers to the compensatory strategy of serial reading, usually found in these patients. Serial processing of letters leading to a mild word length effect may occur in other neurological conditions.

Two observations have been interpreted as evidence for serial letter processing: Some patients read aloud each individual letter and, thus, explicitly assemble the target word. Obviously, the more letters there are in a word the slower the response. Other patients may read silently but they also exhibit a significant word length effect (WLE). The WLE refers to the observation that reading speed for single words depends on the number of letters with a monotone increase of response time across increasing word length. The WLE, along with better preserved writing, is pathognomic for the disorder [1]–[2], [7]–[8]. It varies considerably between patients [9] but contrasts sharply with unimpaired readers who usually exhibit no or negligible length effects [10]–[13].

Some of the few available eye movement studies have provided independent support for letter-by-letter reading. Rayner and Johnson [14] observed that a pure alexic reader's eye movements closely resembled unimpaired readers who could only view one letter at each fixation. Pflugshaupt et al. [3] also reported that patients with pure alexia showed at least one fixation per letter in a word. In addition when reading text, they have been found to exhibit longer average fixation, shorter saccades, and more regressions [15].

One complication for the previously mentioned results is that pure alexia usually goes along with defects in the right visual field (visual field defects, VFD), usually homonymous hemianopia. Although there are rare reports of pure alexia patients without visual field defects (e.g., [12], [16]–[18]) the majority of pure alexic patients also suffer from a right VFD. In a review by Leff et al. [19], this applied to more than 90% of patients. Obviously, a right VFD such as hemianopia interferes with visual processing and, thus, affects reading [20]–[22] as the perceptual window to the right of fixation is unavailable and saccades can be programmed less efficiently [23]–[24]. Hemianopic patients, thus, exhibit shorter saccades to the right as well as more and longer fixations [3]. They have also been shown to exhibit a word length effect albeit considerably smaller than pure alexic patients.

There is both experimental as well as anecdotal evidence that the VFD contributes to the reading pattern of pure alexia: In a recent study, Starrfelt et al. [8] investigated word and nonword processing in four patients with pure alexia. All four were better at processing words in comparison to nonwords but this was related to the size of their VFD. The authors argued that the VFD contributed to the patients' efficiency of word processing. In addition, letter substituation errors at the end of words suggest impaired letter processing especially in the right visual field. For example, one of the participants of the present study, DH, read the words “HELD” (‘hero’) and “KONZERT” (‘concert’) as “HELM” (‘helmet’) and “KONZEPT” (‘concept’). While confusion of “R” and “P” is ambiguous and could be due to the similar shape of the letters, the former error is more difficult to interpret as a confusion of letter shape.

Several authors have identified criteria to distinguish between patients with hemiaopia and those suffering from pure alexia and VFDs. Leff and Starrfelt [1] as well as Sheldon et al. [12] suggested that a WLE of more than 160 ms per letter would indicate pure alexia. Sheldon et al. also compared pure alexic readers with unimpaired readers who read with a simulated VFD. They could demonstrate that both response times and number of fixations in single word reading were increased in pure alexia in comparison to hemianopic alexia and controls with simulated VFD. Thus, it is clear that hemianopia alone does not cause pure alexia. Rather, pure alexic patients suffer from an additional deficit which has been suggested to consist of an impaired visual input lexicon [25] or a general impairment of visual processing [8], [26]–[28].

The present study set out to compare the reading of pure alexic readers with the reading of unimpaired individuals with a simulated VFD. The study makes use of an experimental paradigm recently introduced by Sheldon et al. [12]. However, these authors had limited their analyses to response times and number of fixations, which are highly correlated. Eye tracking methodology, however, provides additional measures such as fixation duration, saccade amplitude, and sequences of saccades [24]. Previous studies have argued that pure alexia reflects letter-wise processing [3], [14] but the serial, “letter-by-letter” strategy has not been subject to empirical study. This becomes even more relevant when considering that some pure alexic readers read silently and when considering the variable word length effects in hemianopic and pure alexia [1], [9], [19]. For example, the letter-by-letter reader DS, reported by Behrmann and Shallice [29], exhibited an increase of 108 msec per letter, much less than the suggested 160 msec [1], [12].

In addition, eye movements may also serve to assess the fixation pattern in patients with atypical aetiology. One of the alexic participants of the present study, DN, had suffered from an intracerebral hemorrhage unlike the more typical ischemia in the left posterior cerebral artery, and his lesions were associated with an atypical visual field defect. Although atypical aetiologies as well as atypical visual field defects have been described [3], [7] the present study documents differences and similarites between patients. The present study will address these questions by investigating eye movements in single word reading of pure alexia patients and controls with simulated VFD (cf. Sheldon et al. [12]).

## Patient Information

### Clinical background and linguistic background

**Figure 1:**
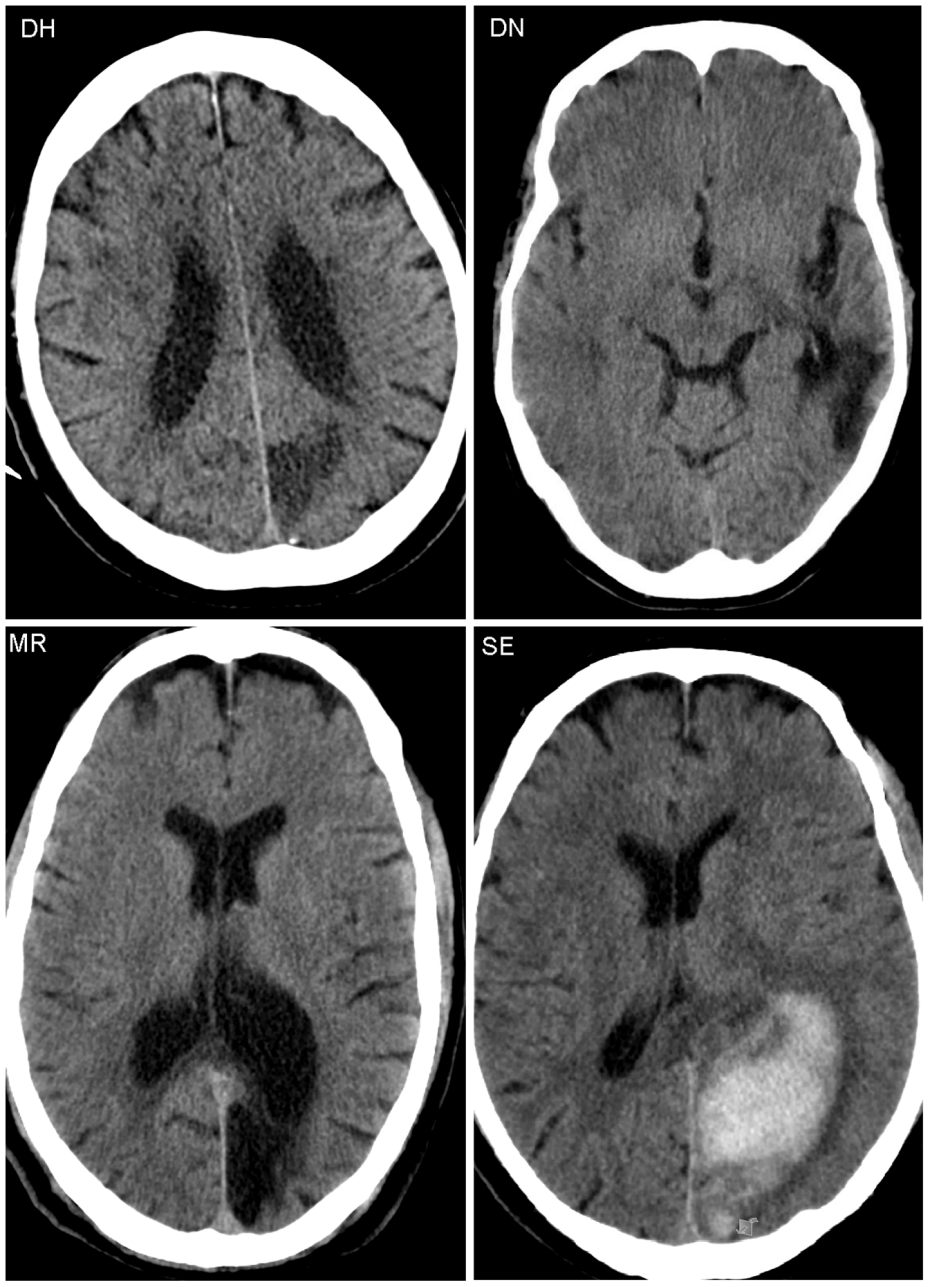
CT scans of the alexic participants.

**Figure 2:**
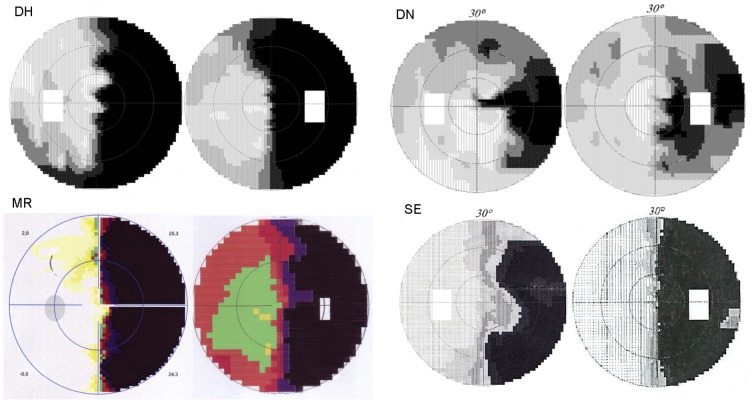
Results of 30° perimetry (Octopus) for the alexic subjects.

Details of the medical records of the four alexic participants can be found in **Table 1**. This table also provides information on the participants' language skills, based on the assessment with a standardized neurolinguistic test battery (“LeMo”, [30]). All four participants complained about mild word-finding difficulties [31] which could be formally verified for DN and DH. DN had a more complex medical record with a myocardial infarction in 1994, a CVA in the left MCA in 2000 and an intracerebral hemorrhage in left temporal areas in 2007. The stroke in 2000 had caused mild symptoms of aphasia from which, according to his relatives, DN had recovered. He nevertheless produced occasional phonemic and paragrammatic errors as described in a previous study [32]. The hemorrhage from 2007 had resulted in his reading impairment and a left sectoranopia suggesting involvement of the thalamus [33]–[34]. The lesions also affected the inferior temporal gyrus and fusiform gyrus around the left occipitotemporal sulcus reaching up into the adjacent parietal and occipital lobe. CT or MR scans of the four alexic participants are shown in **Figure 1**. Visual field defects of DN, DH, and MR were assessed in the Department of Ophthalmology and evaluated by one of the authors (W.L.). SE's perimetry was carried out by a practice-based ophthalmologist. Results of the 30° automated perimetry (Octopus, Haag-Streit, Koeniz, Switzerland) are presented in **Figure 2**.

**Table 1:** Sociodemographic and linguistic description of the alexic participants.

	DN	DH	MR	SE
Age	69 years	76 years	62 years	71 years
Profession	engineer	high school teacher	technician	high school teacher
Etiology	left temporal ICH	ischaemia left PCA	ischaemia left PCA	ischaemia left PCA plus ICH
months post-onset	47	12	21	9
visual field defect (right visual field)	scotoma	hemianopia	hemianopia	hemianopia
Auditory lexical decision (LeMo 5; 73–80^1^)	77/80	75/80	*n.a.*	79/80
Single word repetition (LeMo 9; 37–40^1^)	38/40	36/40	40/40	40/40
Writing to dictation (LeMo 21; 37–40^1^)	38/40	39/40	*n.a.*	40/40
Word-Picture Matching (LeMo 23; 19–20^1^)	20/20	20/20	20/20	20/20
Oral Naming (LeMo 30; 19–20^1^)	16/20	17/20	19/20	19/20

Legend:

1 normal range in this subtest;

ICH = intracerebral hemorrhage;

PCA = posterior cerebral artery;

*n.a.* = not administered.

All subjects achieved formally unimpaired scores in the subtests of two batteries for visual processing (VOSP [35]; BORB [36]). Results of the participants are presented in Table 2.

**Table 2:** Performance on two testbatteries of visual processing.

	*normal*	*DN*	*DH*	*MR*	*SE*
Visual Object and Space Perception Battery (*VOSP*)					
Screening Test (Finding X's)	15–20	18/20	19/20	20/20	20/20
Incomplete Letters	16–20	19/20	20/20	20/20	20/20
Object Recognition	14–20	19/20	20/20	18/20	18/20
Dot Counting	8–10	10/10	9/10	10/10	9/10
Position Discrimination	18–20	19/20	20/20	20/20	19/20
Cube Counting	6–10	10/10	10/10	10/10	9/10
Birmingham Object Recognition Battery (BORB)					
subtest 2 – length match	24–30	24/30	25/30	27/30	24/30
subtest 3 – size match	23–30	26/30	29/30	28/30	24/30
subtest 7 – minimal feature match	19–25	25/25	25/25	25/25	24/25
subtest 8 – foreshortened match	16–25	25/25	24/25	25/25	25/25
subtest 10 – Object Decision A easy	24–32	30/32	31/32	31/32	30/32
subtest 10 – Object Decision B hard	16–32	31/32	29/32	28/32	28/32

### Assessment of reading, repetition and writing (same word list)

The same set of words was presented for reading aloud, writing to dictation, and repetition on three different occasions. Administration of the three tasks was separated by at least four weeks to avoid training effects. The set consisted of 25 three-, 25 five- and 25 seven-letter nouns matched for frequency and concreteness. The WLE was assessed with an analysis of variance. Results for reading are presented in **Table 3**, along with response times of five unimpaired controls (mean age 60 years, 45–76 years). Response times were measured to the closest milisecond by means of a voice key. Trials with voice key failures, reading errors, and hesitations were discarded (2%). Results for the patients' repetition and writing of the same words is listed in **Table 4** showing preserved repetition of the stimulus words as well as better preserved writing.

**Table 3:** Response times and the WLE (in ms).

	3 letters	5 letters	7 letters	WLE/letter
DH	5642	9220	9670	1007
DN	2258	5075	8269	1503
SE	1886	2756	3258	343
MR	3568	4962	6143	644
Control 1	505	505	576	18
Control 2	475	501	477	1
Control 3	429	411	430	0
Control 4	477	479	502	4
Control 5	481	489	473	−2

**Table 4:** Writing and repetition of the set of 75 words along with types of spelling errors.

	*DN*	*DH*	*MR*	*SE*
Repeated correctly	71	72	75	74
Written correctly	68	67	69	71
PPEs in writing	3	6	6	4
Other writing errors	4	2	0	0

**Figure 3:**
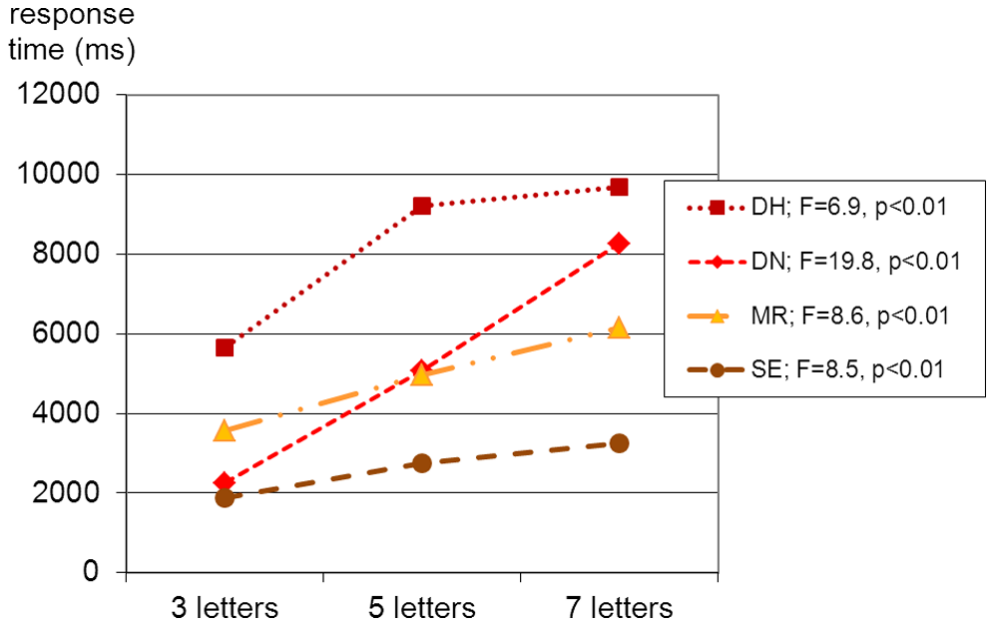
Alexic participants' length effects in word reading (in milliseconds).

**Figure 3** presents the average reading times for the three groups of words along with the respective F-value of the analysis of variance. The subjects differed in their reading behavior: DH, MR, and SE usually read silently until they produced a response (e.g., [start of presentation] “… [8.5 seconds] … catalogue!”). They reported that they had to process the letters individually to come up with the overall response. In contrast, DN did not read silently but overtly produced fragments, comparable to an effortful conduite d'approche (e.g., [start of presentation] “ca cata cala catal cala calatog cala cata cata catalogue, yes, catalogue, gosh, that was quite a piece of work!”). Beginning of presentation as well as the responses of the subjects were digitally recorded, and the time between onset of presentation and the beginning of the first complete (correct) response was determined. Each alexic participant exhibited a length effect significant at the 1% level.

## Experimental Studies

### Ethics statement

The study was approved by the local institutional review board (Ethikkommission der Universität Freiburg). Written informed consent was obtained from all participants. The patients mentioned in this manuscript have given written informed consent (as outlined in the PLoS consent form) that details about their medical history are published.

### Control subjects

**Figure 4:**
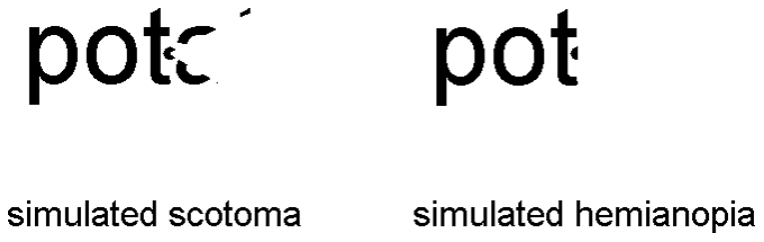
Sketch of the simulated VFD when fixating letter “a” of “potato”.

There were nine control subjects reading lists of words without and with simulated hemianopia or scotoma. Their mean age was 71.2 years (65–77 years), their educational background was comparable to the alexic subjects. Three control subjects were confronted with a simulated VFD comparable to DN's sectoranopia, six subjects had a simulated VFD equivalent to right-sided homonymous hemianopia (RHH)(**Figure 4**). None of the controls had neurological or psychiatric impairments, and all had normal or corrected-to-normal vision. The experimental session lasted about an hour, subjects were reimbursed for participation.

### Apparatus

The experiments were set up with the “Experiment builder” software (SR Research Ltd., Mississauga, Canada). The eye tracker that was used for DN, MR, SE and the controls was an EyeLink 1000, tracking system with a head rest. It offers an accuracy of 0.25° to 0.5° of the visual field, and a temporal resolution of 1000 Hz. For DH, a head-mounted EyeLink II tracker was used. DH tended to squint his eyes during reading which affected the corneal reflection and led to track losses. Therefore, tracking was carried out based only on the position of the pupil. Accuracy was still around 0.5° of the visual field in average. The EyeLink II system has a temporal resolution of 500 Hz when tracking in pupil-only mode and corrects for the head position and rotation of the participant. Both eye trackers are from the same company and make use of the same software, camera settings etc. making the results comparable.

DN, MR, SE and the controls were presented the stimuli on a 20-inch TFT flat screen. The distance between the EyeLink 1000 head rest and the screen was 60 cm. DH was presented the stimuli on a 19-inch CRT screen. The distance between DH's eyes and the screen was between 50 and 60 cm. Distance variations were compensated by the tracker system.

The gaze contingent white mask blocked view to the right of a subject's current fixation. Gaze positions were acquired at a temporal resolution of 1000 Hz. The position of the mask was updated within around 15 msec, dependent on the current processing load of the tracker system and the next screen update cycle.

### Experiment 1

#### Materials

Two lists were created of 110 words each. The words in the two lists were matched for CELEX word frequency [37], lexical orthographic neighbors, length, and concreteness (**Table 5**). Words were presented individually in 44pt. Arial on the center of the screen. Words were between three and nine letters long and were printed in lower case letters with initial capital letter.

**Table 5:** Features of words in the two lists of words (standard deviation).

	List A	List B	t-value	p-value
CELEX frequency	550 (1436)	526 (790)	<1.0	p>0.20
length	5.1 (1.4)	4.8 (1.3)	1.4	p>0.15
lexical neighbors	2.6 (2.6)	3.1 (2.9)	−1.5	p>0.13
number of abstract words	45	45		

#### Procedure

Prior presentation, a fixation point appeared on the screen which the participants had to fixate, the word then appeared right to their fixation. As in the background experiment, the time between the word onset and beginning of the complete correct response was measured.

Control subjects received the information that parts of the visual field were covered and that the cover would move with their eye movements. This was necessary since an additional control subject, excluded from the analyses, did not recognize the beginning of the presentation and would remain on the fixation spot for several seconds when not prompted to move his eyes. In order to avoid biasing the controls' reading due to their experience with a sVFD, the baseline condition without a sVFD was presented first, immediately followed by the VFD condition.

#### Results

The present study analyzed reading latencies, number of fixations, mean fixation duration, number of fixations per letter position as well as serial order of fixations. Fixations below 80 milliseconds were excluded from the analyses.

**Table 6** provides the average response latencies for controls without and with the simulated visual field defect along with the alexic readers' response latencies. An analysis of variance with “type of VFD” (RHH versus scotoma) and “VFD present/absent” revealed a highly significant main effect of the simulated VFD (present vs. absent; F_(1, 7)_ = 15.1, p<0.01). There was a trend towards faster responses with simulated scotoma and slower reading with simulated RHH (F(1,7) = 4,6, p = 0.07) and a trend of an interaction (F(1,7) = 4.4, p<0.08) as the controls with RHH were affected more than the controls with scotoma. When controls with sVFD and the alexic readers were compared, significantly slower latencies for the alexic readers (4899.7 versus 1601.4 msec.; t_(3.2)_ = 3.75, p<0.04) was observed.

**Table 6:** Mean reading times, fixations, and fixation durations (plus standard deviation).

	errors	reading time in msec	no. of fix.	fix. durat. in msec
controls baseline	0.3 (0.7)	873 (65)	2.7 (0.3)	291 (56)
controls with sim. VFD	4.8 (4.2)	1601 (474)	3.7 (1.6)	313 (38)
controls with scotoma	5.7 (4.4)	1204 (193)	2.8 (0.7)	313 (53)
alexic reader DN	2	3538 (2157)	11.0 (1.8)	240 (133)
controls with RHH	4.3 (4.5)	1800 (449)	4.2 (1.7)	313 (35)
alexic reader MR	9	6870 (2152)	14.1(4.8)	414 (184)
alexic reader SE	3	3356 (1051)	8.5(3.8)	312 (144)
alexic reader DH	0	5836 (2225)	11.8 (4.6)	379 (172)

The controls exhibited a non-significant increase in the number of fixations when confronted with a sVFD (3.7 versus 2.7 fixations; F(1,7) = 2.3, p>0.15). There was no effect for type of VFD (F(1,7) = 1.56, p>0.25) and no significant interaction (F(1,7) = 1.45, p>0.25). A comparison of controls with sVFD and the alexic readers revealed a significant difference for the number of fixations (3.7 versus 11.4; t_(11)_ = 7.1, p<0.01). The controls' mean fixation duration increased under the condition of the VFD (312.6 versus 290.5) but this increase was not significant (t<1.1). Likewise, there were no differences in fixation durations for the type of VFD and no difference between the controls and the alexic patients at the group level (t<1.0). DN's fixation durations were shorter than the controls' with a marginally significant result (t = 1.6, p = 0.07). DN's fixation durations were shorter than all alexic participants' (all *t*'s>10.0, *p*'s<0.01).

**Figure 5:**
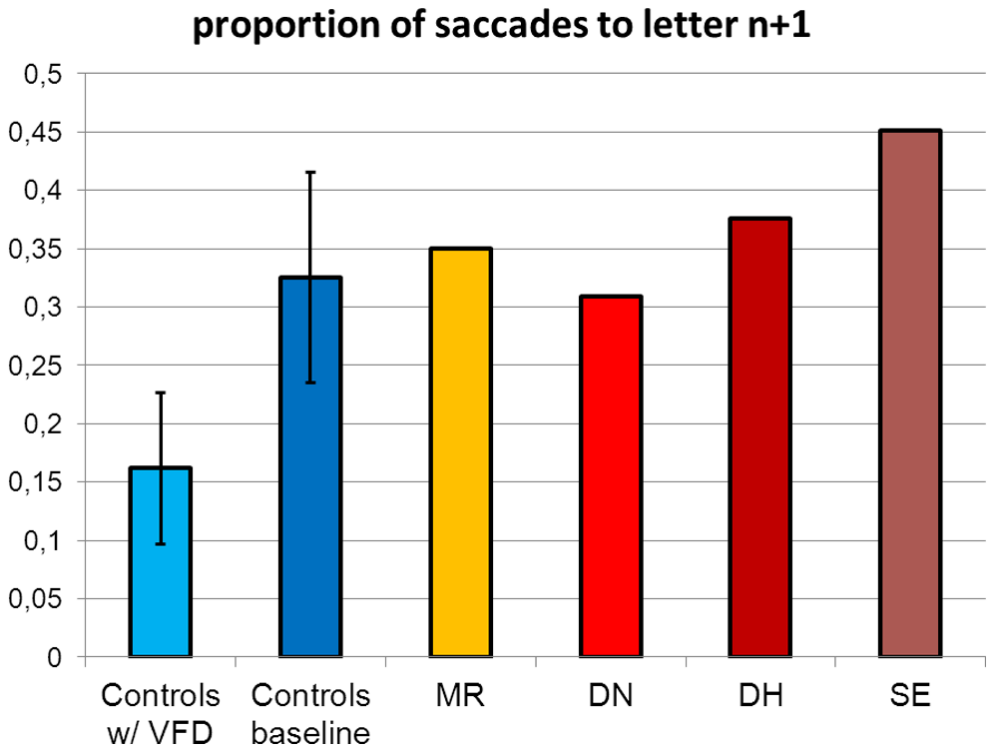
Proportion of saccades to letter n+1.

The proportion of saccades moving to the next letter in relation to all saccades were calculated for each participant. In the baseline condition, 33% of saccades were directed towards the next letter while in the VFD condition, only 16% were aimed at the next letter (**Figure 5**). The difference was significant (t_(8)_ = 5.2, p<0.01). The difference between the patient group and the controls with sVFD was significant (t_(11)_ = 5.6, p<0.01). In the analysis of saccades, controls with sVFD exhibited greater amplitude than the alexic readers (3.7±1.3° versus 2.0±0.1°) with only minimal variation within the alexic group (MR: 1.9°; DN: 2.0°; DH: 2.1°; SE: 2.1°). The difference between controls and patients was significant (t_(11)_ = 2.65, p<0.05).

**Figure 6:**
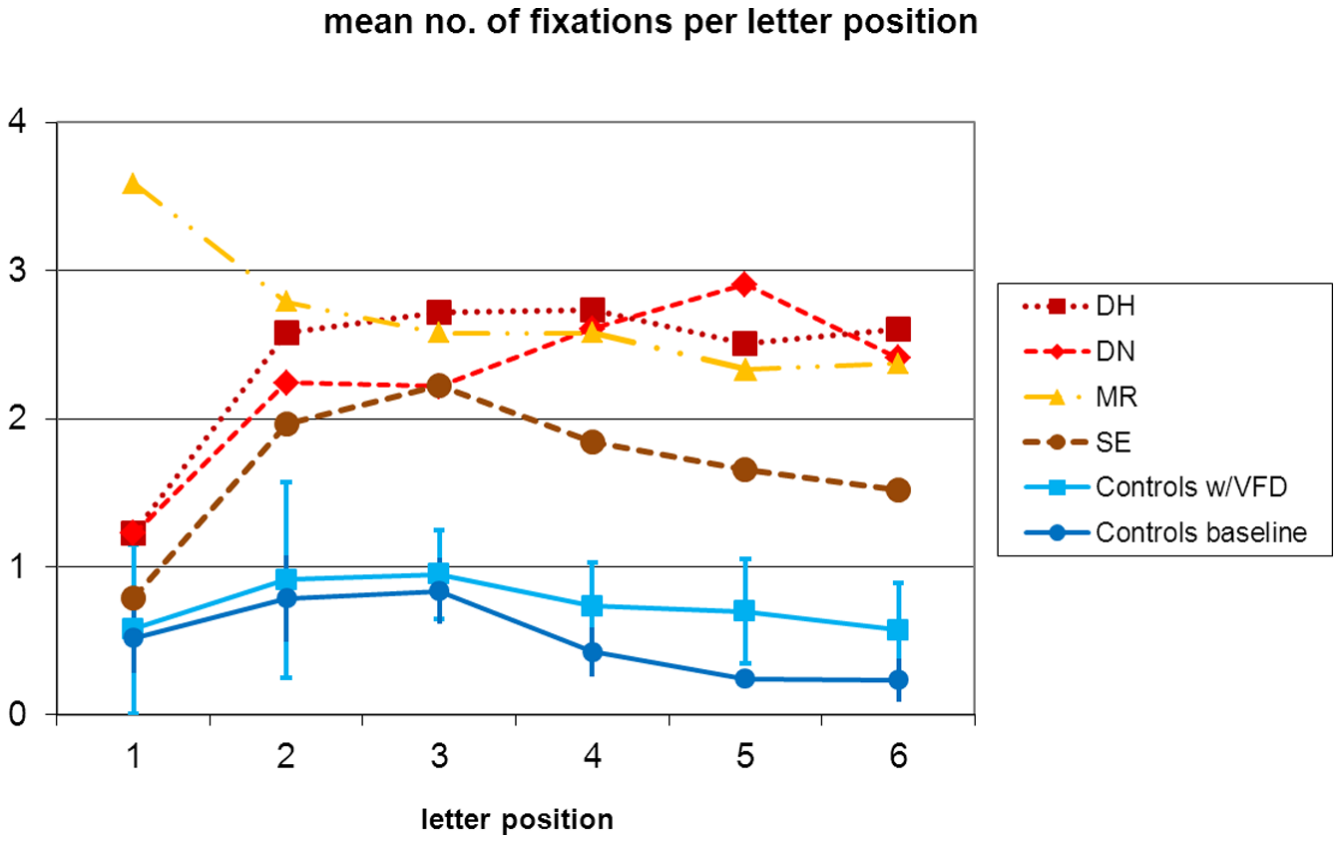
Fixations across letter positions.

In an analysis of mean number of fixations across letter positions 1 to 6, the effect of letter position was significant (F_(1.5, 40)_ = 5.5, p<0.05). There also was a main effect for viewing condition with more fixations in the VFD condition (F(1,8) = 6.3, p<0.05) while the interaction missed significance (F(5, 40) = 2.1, p<0.10). In the comparison of controls with sVFD and the alexic readers, the effect of position missed significance due to a violation of sphericity (F(1.5, 55) = 2.6, p<0.11), the interaction missed significance (F<1.0), but the difference between the controls and the alexics was significant (F(1,11) = 57.7, p<0.01). **Figure 6** suggests that controls exhibited a top-down effect with more fixations on word-initial letters, most likely because the number of lexical candidates can be narrowed down after more letters have been identified. From visual inspection, this pattern was preserved only in alexic readers SE and MR but less so in DN and DH.

#### Discussion

The first experiment aimed at a description of eye movements in four patients with pure alexia in comparison to eye movements of controls with sVFD. It has been reported previously that a sVFD did affect control participant's eye movements and response times in single word reading but that the resulting pattern were different from pure alexia [12]. The same has been found in the present experiment showing that an artificial VFD impaired participants's response times and number of fixations but not as dramatically as seen in pure alexia.

Unlike Sheldon et al. [12], who had focussed on the effect of length on response times and number of fixations, we assessed additional variables (fixation duration; saccade length, serial fixation pattern). Only two of four alexic patients exhibited longer fixation durations than the controls which was unexpected given previous reports of differences [3], [15]. These reports, however, had assessed reading of sentences or paragraphs, not single words. In the present experiment employing single word reading, no consistent difference emerged for fixation durations which can be attributed to the difference in information uptake between the two groups. The pure alexic readers did, indeed, fixate each individual letter at least once while in contrast, controls exhibited fewer fixations and, thus, exhibited processing of multiple letters upon each fixation. Therefore, the information processed upon each fixation may differ. The difference between pure alexic readers and controls with sVFD is mainly in the response times and the number of fixations.

In contrast, there was a significant difference with regard to the serial letter processing strategy, known to be typical for pure alexia. “Letter-by-letter reading” proper is reflected by a higher proportion of saccades directed towards the adjacent letter to the right. This serial reading strategy could not be observed in unimpaired readers with sVFD. In contrast, the proportion of serial fixations was actually higher in the baseline condition and decreased in the sVFD condition. If letter-by-letter reading could be provoked by an artificial VFD in the controls, they should exhibit a similar proportion of fixations directed towards the next letter, even if they exhibited fewer overall fixations. Contrary to these intuitions, the proportion of saccades directed to the next letter was actually lower in the controls reading with sVFD than in the baseline condition! This suggests that the letter-wise reading behavior in pure alexia is independent of the VFD. The higher serial fixation strategy in the pure alexic readers was associated with smaller saccade amplitude.

Finally, with respect to the distribution of the fixations across the words, there was a significant effect of letter position for the controls suggesting fewer fixations over the course of the words, both in the baseline condition as well as with simulated visual field defect. This pattern was present only in one alexic participant, SE. MR exhibited a top-down effect over the course of letter positions but with an atypically high number of fixations at the initial letter. The other two alexic readers, DH and DN, did not show a top-down effect but a plateau (DH) or even an increase of number of fixations across letter positions (DN). Top-down effects during reading, thus, could be observed in only two of the four alexic readers. The next experiment assessed more systematically the difference between four- and six-letter words. In addition as a further variation, words were presented in the preserved left visual field.

### Experiment 2

#### Materials

Two lists were compiled consisting of 25 four- and 25 six letter words each. Healthy controls read both lists, one without a sVFD, the second with sVFD. Four-letter words in both conditions were matched for frequency (dlex [38]), summed bigram frequency, and neighbors (Coltheart's N; **Table 7**). The same applied to the six-letter words of both lists. Across length (four versus six letters), the lists were matched for word frequency. The alexic subjects read one of the lists.

**Table 7:** Linguistic features of the words used in Experiment 2.

	List 1		List 2	
	4 letters	6 letters	4 letters	6 letters
dlex word frequency	3976.4	3877.5	3418.9	3750.3
lexical neighbors	33.6	12.8	33.9	13.3
summed bigramm frequency	606844.6	1300450.9	610264.1	1358414.3

#### Procedure

Words were read individually on the screen. A fixation point to the right of the word would preceede each trial. Upon fixation of that point, presentation of the word was started accompanied by a short tone. The alexic subjects read one of the lists aloud. Control subjects read both lists. They started with the ‘unimpaired’ condition with free viewing followed by the condition with the simulated visual field defect, the participants were granted breaks between the two conditions. If necessary, recalibration was done after the breaks. There were three practice trials to familiarize subjects with the simulated visual field defect.

#### Results

Average reading latencies for all participants are presented in **Table 8**. The simulation of a visual field defect in the controls led to an increase of reading times: In a 2×2 analysis of variance (VFD present/absent; word length), there was a significant main effect of reading condition (slower reading with simulated visual field defect; F_(1,8)_ = 23.0, p<0.01). There was no effect of word length (F<1) and no interaction (F<1). With simulated paracentral scotoma, there was a difference of about 47 milliseconds between six- and four-letter words. With simulated hemianopia there was a numeric advantage for six- over four-letter words (36 miliseconds). Both differences, however, were non-significant (t<1.0). The type of the VFD did not affect reading times (F<1.0), so the controls will be evaluated without further reference to subgroups.

**Table 8:** Response latencies in msec and fixations in Experiment 2 (plus *standard deviation*).

	complete list (4 and 6 letters)	four-letter words	six-letter words	t	p
*mean reading times*					
Controls					
without VFD (list 1)	901 (*87*)	901 (*103*)	900 (*73*)	.097	n.s.
with simulated VFD (list 2)	1446 (*374*)	1450 (*446)*	1441 (*313*)	.115	n.s.
subgroups of controls (list 2)					
with simulated RHH	1515 (*388*)	1533 (*459*)	1497 (*346*)	.371	n.s.
with simulated scotoma	1306 (*329*)	1282 (*452*)	1329 (*252*)	.394	n.s.
alexic participants (list 2)					
DH	6771 (*3489*)	6113 (*2499*)	7457 (*4234*)	1.36	= 0.18
DN	4927 (*3387*)	3207 (*1714*)	6575 (*3788*)	3.89	<0.01
MR	5618 (*2592*)	4588 (*1326*)	6694 (*3137*)	3.02	<0.01
SE	3223 (*760*)	3041 (*711*)	3391 (*779*)	1.62	= 0.12
*mean number of fixations*					
Controls					
without VFD (list 1)	2.8(*0.4*)	2.6 (*0.3*)	3.1 (*0.4*)	15.6	<0.01
with simulated VFD (list 2)	3.7 (*1.4*)	3.3 (*1.5*)	4.1 (*1.3*)	4.5	<0.01
alexic participants (list 2)					
DH	13.2 (5.6)	12.3 (*5.4*)	14.2 (*5.8*)	−1.2	n.s.
DN	16.8 (*11.2*)	10.8 (*5.1*)	22.5 (*12.4*)	−4.3	<0.01
MR	14.0 (*5.9*)	11.5 (*2.9*)	16.7 (*7.0*)	−3.3	<0.01
SE	9.0 (*2.3*)	7.9 (*2.1*)	10.0 (*2.1*)	−3.5	<0.01

The increase in reading times did not match the latencies observed for alexic readers (cf. **Table 8**). Along with a significant main effect for word length (F_(1, 11)_ = 18.5, p<0.01), there was a significant group effect (alexics slower than controls with sVFD, F_(1, 11)_ = 53.05, p<0.01) and a significant interaction (F_(1, 11)_ = 18.8, p<0.01) suggesting a larger length effect for the alexic participants.

**Figure 7:**
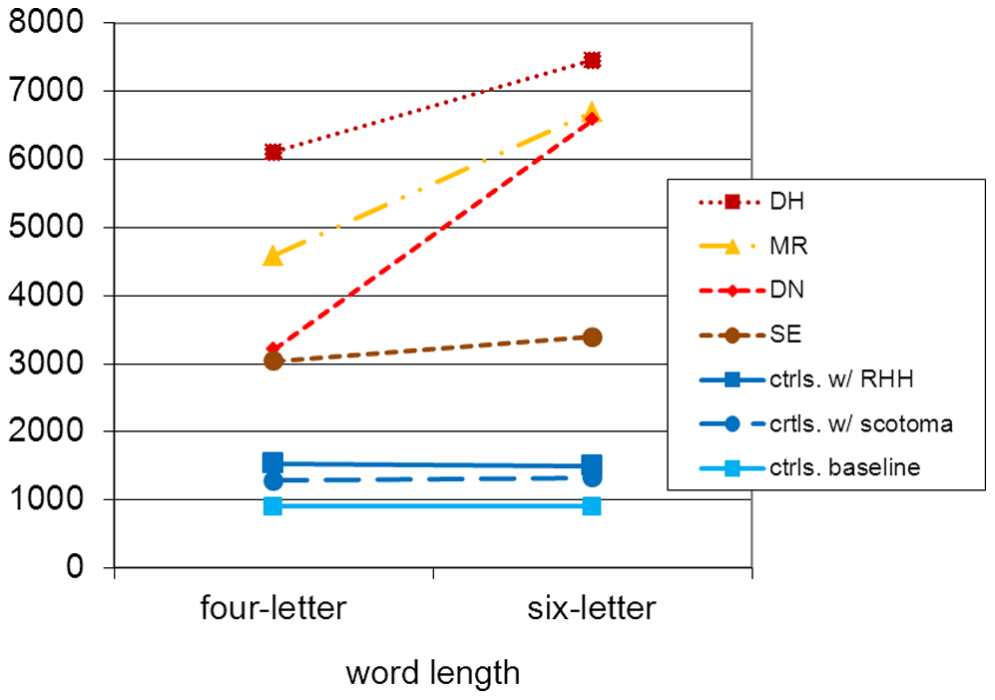
The length effect for response times (in msec.).

At the level of the individual alexic readers, the length effect was considerable but missed significance in two alexic participants. The difference of 1300 milliseconds for DH missed significance, as did the difference of roughly 350 miliseconds for SE. Variability within condition is the most likely reason. Still, these increases exceed the controls' increase and fall within the range of other alexic readers [1]–[3]. For DN and MR, the increase was significant. **Figure 7** allows for a visual comparison of the length effects in controls' and the alexic readers' overt responses.

**Figure 8:**
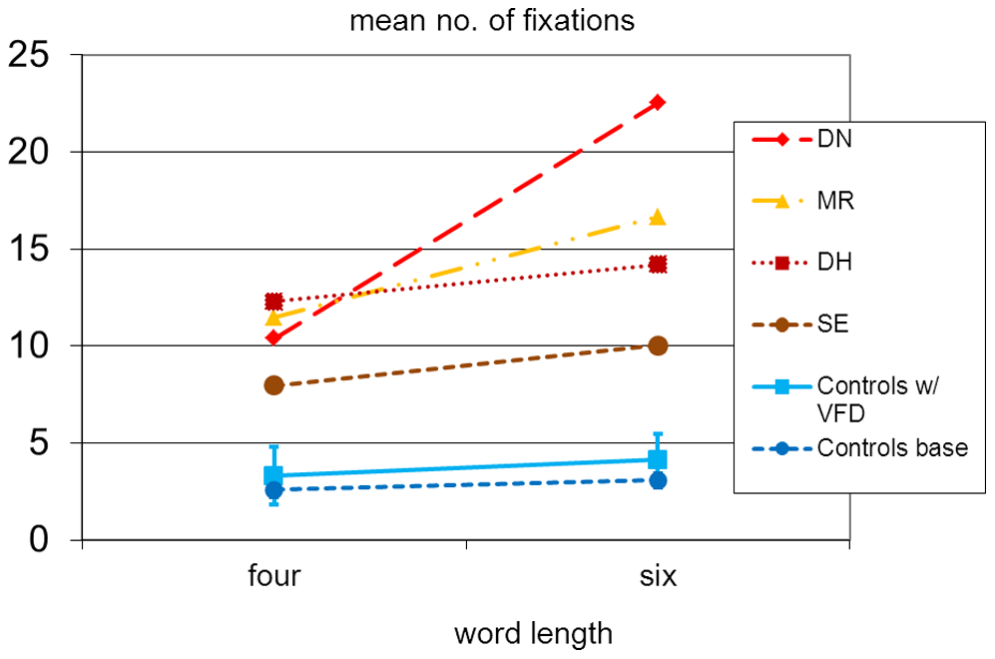
The length effect for the number of fixations.

A slightly different pattern emerged in the analysis of fixations (cf. **Table 8**, **Fig. 8**). For the controls, a 2×2 ANOVA with condition (VFD absent/present) and word length as factors revealed a marginally significant main effect of the sVFD (F(1,8) = 4.7, p = 0.062) and a significant length effect (F(1,8) = 41.8, p<0.01). There was a minimal length effect already in the baseline condition with an increase of 0.5 fixations for the additional two letters. With sVFD, longer words received an average of 0.8 additional fixations. Critically, however, the interaction was not significant although there was a trend (p = 0.09) to be discussed later. The comparison of the two groups (alexic readers versus controls with sVFD) revealed significantly more fixations for the alexic subjects (F(1,11) = 61.9, p<0.01), a significant length effect (F(1,11) = 16.3, p<0.01) and a significant interaction (F(1,11) = 8.7, p<0.02).

When the direction of fixations were assessed the same picture emerged as in Experiment 1: The alexic participants had a larger proportion of fixations directed towards the next letter to the right than controls reading with a sVFD (0.37±0.06 versus 0.17±0.05; t_(11)_ = −6.1, p<0.01).

#### Discussion

Experiment 2 replicated the findings from Sheldon et al. [12] and Experiment 1 that the sVFD did not cause a reading deficit comparable to pure alexia. The experiment again revealed an increase of reading time and number of fixations when a VFD was introduced. This increase was significant for most of the individual control participants and for the whole group. However, this increase was less than one fixation on average and was, by no means, comparable to the alexic readers' response latencies and fixations. Even with a simulated VFD, the reading times did not even approach the latencies exhibited by the alexic readers.

The experiment also assessed the length effect by comparing four-letter to six-letter words. If letter-by-letter reading were caused by visual field defects, one would expect a significant interaction between the controls' reading condition and word length in that a stronger length effect should emerge with sVFD. In contrast, there remained significant differences between alexic readers and controls both quantitatively and qualitatively: For the alexic readers, the two additional letters led to an increase of reading time between 350 milliseconds (participant SE) and 2100 milliseconds (participant MR). In contrast, no clear length effect was observed for the controls with sVFD.

The present experiment also used a variation in the presentation mode, namely presentation of the stimulus words in the preserved left visual field. If words appear in the preserved visual field they are completely visible until a fixation is made to the left. If the VFD to the right caused the alexia in the patient group, they should exhibit a similar pattern as the controls with the sVFD. If, however, alexia was caused by an additional deficit, there should be differences between the two groups which, indeed, have been found. Pure alexic readers exhibited a slower and serial reading pattern. This suggests that the disadvantage of pure alexic patients persists even if words are presented in their preserved left visual field. This replicates various behavioral studies that presented words in the left visual field (e.g., [39]).

## General Discussion

A stroke in the left posterior cerebral artery frequently leads to left-sided visual field defects and thus affects the ability to read fluently (e.g., [21]–[22], [40]–[41]). In more severe cases, a peripheral alexia with a slow, letter-wise reading strategy results. Patients suffering from ‘pure alexia’ have been argued to benefit less from rehabilitation than patients with hemianopia [42].

The present experiments compared reading in four patients with pure alexia to reading of unimpaired individuals reading with a simulated VFD. This experimental approach was first introduced by Sheldon et al. [12]. This study as well as the present one are the first to assess eye movements in pure alexic single word reading and, thus, help to bridge the gap between traditional neuropsychological case studies with a focus on word processing, and eye movement research which mainly employs sentence reading.

Sheldon et al. [12] had limited their analyses to response times and number of fixations which are necessarily correlated. More fixations should occur in slower responses. The present study's findings parallel those by Sheldon et al. [12]: The sVFD affected reading times in healthy readers but these ‘impairments’ were not comparable to the reading of pure alexic patients. In terms of reading times, a sVFD led to a mild slowing but latencies were still significantly faster than the alexic readers' mean response times. The response times of the unimpaired readers in the baseline condition were slower than usually reported. For example, Balota and Ferraro [43] reported reading times between 640 and 760 msec for participants of 71 years of age. We suggest that the unfamiliar setting of word reading with the chin resting on a chin rest and our instructions to avoid errors led to an emphasis on accuracy rather than speed.

While the controls exhibited a mean of 3.7 fixations when reading with a sVFD, an increase of one fixation in comparison to baseline, the alexic patients had an average of 11 fixations for words with, on average, five letters (Experiment 1). Thus, on average, the alexic readers fixated each letter twice while there was less than one fixation per letter for the control participants.

Experiment 2 assessed the length effect but also presented target words in the preserved left visual hemifield. Thus, target words were visible initially before any saccade was carried out but this did not alter the basic pattern: The sVFD caused an increase in reading latencies and number of fixations but these still differed significantly from the alexic patients' results. While the controls with sVFD had an average reading time of 1500 ms, the fastest alexic reader, SE, responded in 3200 ms, and the slowest, DH, had a mean response time of 6700 milliseconds. The experiment further revealed that controls with sVFD did not exhibit a length effect comparable to the alexic patients.

As a new observation, however, both experiments revealed a larger proportion of serial, letter-wise fixations in the alexic in comparison to unimpaired participants, even when the latter read with sVFD. The present study, thus, is the first to provide an operationalization of “letter-by-letter reading” proper and to document this serial fixation strategy in pure alexia but not in simulated right visual field defects. It was found that with sVFD, healthy readers exhibited even less of a serial reading strategy. This pattern was observed independently of the overall number of fixations which was significantly higher in the clinical group. With unimpaired view, the perceptual window to the right was large enough, so that fixations on the initial letters were enough to identify the words. In contrast with sVFD, participants had to make wider saccades to place the target word in their preserved left visual field. Controls with sVFD, thus, exhibited wider saccades than the pure alexic patients. These observations are especially relevant when considering that hemianopic alexic patients, too, exhibit a word length effect, that the word length effect in pure alexia varies considerably between individuals (e.g., [2], [9]) and, finally, when acknowledging the diversity of additional visual impairments in pure alexia (e.g., [4], [44]–[46]).

The analysis of the individual pure alexic patients revealed differences some of which were unexpected. First, only two of four patients exhibited an increase in fixation duration in comparison to controls, despite previous reports of such a difference in sentence reading (e.g., [3], [15]). This may, in part, be related to the task of single word reading. While patients fixated individual letters in a letter-by-letter fashion, controls exhibited “parallel” processing of multiple letters, thus allowing for fewer fixations. DN and SE exhibited the shortest fixation durations while MR and DH exhibited longer fixation durations. In both Experiments 1 and 2, they fixated longer than DN and SE. Taken together the data from the four different alexic readers suggest individual differences in adaptation to the deficit.

DN's atypical VFD, a right sectoranopia observed rather rarely [35]–[36], could be demonstrated not to cause his reading impairment as controls with a simulated sectoranopia did not read letter-by-letter. All four patients exhibited comparable saccade amplitudes, and for both variants of the VFD it could be demonstrated that the alexia was not caused by the VFD. The present study, thus, also replicates the findings of Sheldon et al. [12] with a different type of visual field defect.

In general, our results are compatible with the view that pure alexia is not caused by the VFD but that pure alexic readers suffer from an additional deficit. This deficit cannot be identified more closely based on the present results but several previous studies have demonstrated visual impairments affecting processing of any visual stimuli, albeit more dramatically word reading (e.g., [18], [27], [47]). Future studies should investigate the serial fixation pattern in patients with hemianopia. In addition, in line with the suggested visual impairment underlying pure alexia, a future study may demonstrate that patients with pure alexia but not with hemianopia are affected by letter confusability. Finally, patients with pure and hemianopic alexia may respond differently to presentation of words in the left visual field versus central presentation.
